# Reimagining relationality for reproductive care: Understanding obstetric violence as “separation”

**DOI:** 10.1177/09697330211051000

**Published:** 2022-01-31

**Authors:** Rodante van der Waal, Inge van Nistelrooij

**Affiliations:** 36513 University for Humanistic Studies, Utrecht, The Netherlands

**Keywords:** Obstetric violence, midwifery, maternity care, ethics of care, abortion, reproductive justice

## Abstract

*Nursing Ethics* has published several pleas for care ethics and/or relationality as the most promising ethical foundation for midwifery philosophy and practice. In this article, we stand by these calls, contributing to them with the identification of the structural form of violence that a care ethical relational approach to reproductive care is up against: that of “maternal separation”. Confronted with reproductive and obstetric violence globally, we show that a hegemonic racialized, instrumentalized, and individualized conception of pregnancy is responsible for a severance of relationalities that are essential to safe reproductive care: (1) the relation between the person and their child or reproductive capabilities; and (2) the relation between the pregnant person and their community of care. We pinpoint a separation of the maternal relation in at least two discursive domains, namely, the juridical-political and the ethical-existential. Consequently, we plea for a radical re-imagination of maternal relationality, envisioning what care ethical midwifery, including abortion care, could be.

## Introduction

On August 31^st^ 2021, the state of Texas banned abortion after the detection of a fetal heartbeat. On top of that, the law gives citizens the possibility to sue those who “aid and abed” abortion care seekers, such as friends and families, taxi drivers or information providers and medical professionals.^1–5^ It is the most severe abortion ban since half a century in one of the most powerful nations in the world. Shockingly, it will not be enforced by the state who enables it, since that would be unconstitutional. Instead, enforcing the ban will be the responsibility of individual plaintiffs, giving anti-abortion vigilantes the possibility to sue people they do not know or have never met for a chance at a reward of 10.000 dollars.^5^ The ban hence places a bounty on both pregnant people seeking abortion care and those who care for them. The law is not enforced in the traditional way through state, police, patriarchal or medical violence, but the responsibility of patriarchal racialized violence is handed over directly to fellow citizens.^5^ What has been constitutive of both reproductive disciplination as well as reproductive and obstetric violence here clearly comes to the fore in full daylight: the structural destruction of the maternal subjects by severing the relationalities that define them: 1) their relation to their reproductive capacity (reproductive relation); and 2) their relation to their caring community, what we want to call the “midwifery relation”— together we term this double severance of relationality “maternal separation.”^1^

In reproductive policy, law, as well as political discourse and activism concerning abortion, the embryo has been individualized and separated from the pregnant body from the start of the debate about the legalization of abortions, using misleading imaginaries from which mother, womb, placenta, and umbilical cord are erased.^9^ Fetuses are presented in photographs and materialized in puppets as if living on and by themselves, lifting them out of the pregnant body. This separation does not stop with the discourse surrounding abortion. Instead, it continues through the full length of pregnancy. Both the prominent place of the “maternal–fetal conflict” in bio-ethics, which poses the baby as a danger to the mother and the maternal body is vice versa dangerous for the baby, as well as the common view that the baby is “delivered” by a doctor or midwife, reproduce the discursive separation of mother and child instead of understanding childbirth as an active relational cooperation between mother and child.^10–12^ This severance of the reproductive relation also effectuates common forms of obstetric violence, such as “shroud-waving”, where the mother is manipulated into consenting to obstetric policies through the exaggeration of risk concerning the life of her child, playing mother and child off against each other.^13–15^

While discussions about the infant’s health as well as medical ethical dilemmas in situations of maternal–fetal conflict are justified, the primary focus on these questions in medical ethics is problematic. It disguises other, more pressing issues by singling out the “choice” between mother and child, especially because the active agent imbued with this choice is the doctor, or the ethicist, but not the mother herself. Meanwhile, systemic global problems such as reproductive and obstetric violence and racism remain in the periphery of the ethical debate concerning maternity care. An overwhelming focus on both the fetus’ safety and maternal–fetal conflict in medical practice and ethics, as well as on the embryo’s rights in anti-abortion discourse produce a specific biopolitical framework that determines how we look at, think of, experience, and care for pregnancy and childbirth. The emphasis on the maternal–fetal conflict in ethics, obstetric practice, but also in popular culture, as *the* ethical dilemma and medical problematic of pregnancy, not only unjustifiably neglects other issues but also reproduces the severance of the relation between mother and child. Instead of trying to understand the relationality of the reproductive subject and the event of childbirth, or the relationality of fertility and abortion, we continuously re-inscribe both phenomena in a logic of separation.

In this paper, we identify how reproduction is continuously formulated in terms of separation in both the juridical-political as well as the ethical-existential sphere. We follow theorists on maternity and care ethics such as MacLellan (2014) and Newnham & Kirkham (2019), as well as theorists of obstetric violence like Shabot (2020) and Chadwick (2018) who have theorized obstetric violence as a problem of relationality rather than autonomy.^2,3,13,16^ We build further on fundamental insights of feminist care ethicists concerning relationality, dependency, maternity, and vulnerability, such as Joan Tronto (1993), Sara Ruddick (1989), and Eva Feder Kittay (2019 [1999]), as well as the scholarship on relational autonomy (MacKenzie & Stoljar 2000; MacKenzie, Rogers & Dodds 2014).^17–21^ We aim to illuminate how a discursive tendency of separation continues to *inhibit* the relationality that is needed for both relational autonomy as well as care ethics in reproductive care. Consequently, we plea for a relational ethics or praxis regarding abortion, pregnancy, and childbirth care through a re-imagination of the reproductive, maternal, and midwifery relationalities that can challenge and interrupt individualized subjectivity—acknowledging that in the current climate we do not yet know what these relationalities could possibly entail.

## Maternal separation

### The sevarance of the reproductive and midwifery relation

**Figure 1.**
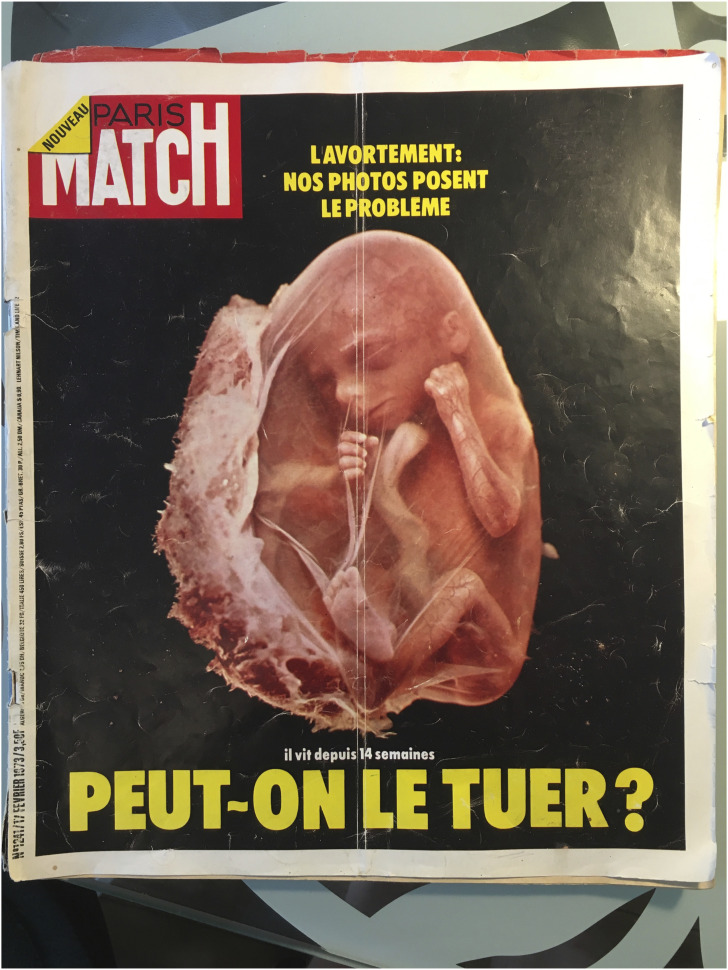
The cover of the Paris Match in February 1973.

In the 1960s in France, Annie Ernaux has an illicit abortion on a cold winter night, the subject of her novel *The Happening* (2000). It is still years before abortion was legalized in France in 1975 and the *Paris Match* ran the cover story “Can we kill him?” in 1973, featuring a photo series of embryo’s floating in empty space (Figure 1).^9^ Ernaux dedicates her book to the woman who performed her abortion, Madame P-R, and to all the women who had helped her along the way—all of whom could now be sued in Texas. Although the society in which she lived was determined to separate her from her community of care, Madame P-R made a deep impression on Ernaux, however meager the actual care she gave was. The relationality of this illegal network of women made what Ernaux calls “the world,” possible:

*I have never stopped thinking about her. Involuntarily, this avaricious woman – whose flat was nonetheless poorly furnished – wrenched me away from my mother and into the world. She is the one to whom this book should be dedicated.*

*[…]*

*Now I know that this ordeal and this sacrifice were necessary for me to want to have children. To accept the turmoil of reproduction inside my body and, in turn, to let the coming generations pass through me*.^22^

Her abortion transformed her into a relational subject, forever inscribed by the relation to the women who helped her and gave her the world, and by the abortion that gave her the possibility to make the relation of reproduction her own and hence accept the “turmoil of reproduction” inside her body. What this quote illustrates, is that in order to be able to relate to one’s fertility on one’s own terms, there must be space for both autonomous decisions and meaning-making practices regarding the relational possibility of another within oneself. This, in turn, is only possible within a midwifery relation, i.e., a relational community of care—exactly those relations that the state of Texas is trying to sever.

When we switch our lens to maternity care, we see that the age-old form of caring for birth, midwifery practice, is also based on these two relationalities, namely, the relational perception of mother and fetus, and the relation between mother and midwife.^23–25^ A long-term commitment between mother and midwife and a focus on physiological birth and the relational nature of pregnancy are the essence of the art of midwifery. In most Western countries however, midwifery became appropriated into the obstetric institution, even when midwives work independently. Midwives must continuously relate to and negotiate with the obstetric institution that delivers the dominant discourse and hegemonic epistemology, and functions upon a paternalizing responsibility over the mother and the instrumentalization of the maternal body in favor of the fetus’ safe passage.^6^ As a result, midwives are being torn between their relational ideals and the reality of having to work in a system characterized by protocols, over-medicalization, time-pressure, high workload, and administration.^3^

The seperation of mother and child by the obstetric care provider has its consequences on the relationality between mother and child during pregnancy and birth.^18^. The midwife or obstetrician has the lead in delivering the baby and seems to know not only more about the condition of the child but also about what is best for the child. Simultaneously, the mother is constituted as a complicating, instead of enabling, factor in the process of childbirth, whose main role is to somehow get through the painful event in a docile manner.^26–28^ This leaves the mother cut loose from the status of an active subject whereas she should have been the main active, relationally embedded, subject of reproduction. Disabling the maternal subject to make her own choices actively writes her subjectivity in non-maternal and non-relational terms, making autonomy impossible. Obstetric violence, then, can be understood on a more structural level as “maternal separation” where the mother is denied relationality both with her child (the reproductive relation) and with her community of care (the midwifery relation).

The illegal abortion of Ernaux gave her “the world” through the relationality with the women who cared for her, and through the autonomous experience of the fecundity of her body. However, legal, institutional, political and ethical spheres of today’s society continue to sever these relationalities, just as in Ernaux’s time. A good abortion, pregnancy, childbirth, and parenthood are dependent on an intact relationality between the reproductive subject, the child and a caring community wherein someone can take up a midwifery role. This double relationality is vital for pregnant people to know how to birth and to trust the process of birth, as well as to know how to accept “the reproductive turmoil” inside their bodies and be able to have an affirmative experience of their abortions. The structural tendency to separate reproductive subjects from their caring relations and reproductive capacity is increasing in anti-abortion stances globally, and in the continuous perseverance of obstetric violence and paternalizing care that induces trauma in these relations and inhibits relational care for and from the maternal. Below, we will discuss the continuous separation of the maternal in at least two discursive domains, namely, the juridical-political and the ethical-existential.

### The juridical-political configuration of the maternal in opposition to “Life”

Reproduction is embedded in a societal and political context in which abortion is legally limited across the globe^2^ and in which the maternal is often framed as being in opposition to the interests of the fetus, the child, or even “Life” itself. “Pro-life” anti-abortion activists gain more and more ground in the Western world, in the US, across Europe, to which the European party ECPM attests.^3^ Framing maternal subjectivity as a risk to “Life,” while the maternal is actually a potential source of life, and the acceptance, or allowance, of this discourse as a valid point of discussion in mainstream politics, is the current-day articulation of the traditional grip of nation-states over people’s reproductive bodies. It repeats the separation of relationality between the mother and what she reproduces, making the child into a separate entity that must be protected from the mother.^7,29^ In the Netherlands, for instance, abortion is still part of criminal law and the recent proposal to allow deceased unborn children to be registered as deceased citizens is being used by Christian parties to argue for the rights and personhood of embryos and fetuses.^30^

Even in countries where abortion is legal, reproductive subjectivity remains a taboo, imbued with shame and a sense of irresponsibility when one wants to make use of one’s right to self-determination. Schrupp (2019) refers to the lawyer Nina Strassner who points out this double standard when arguing that a pregnant woman who says “I am unwantedly pregnant, I do not want to birth this fetus” commits an injustice,^31^ but a person who says “I do not want to donate blood, even when someone next to me will die and I would have saved his life with my blood,” clearly falls under the right to self-determination.^31^ The stigma of injustice that clings to maternal agency reproduces a certain conception of pregnancy. The reproductive relation no longer belongs to the maternal, but their reproductive capacity is turned against them by taking away their possibility to take responsibility. Establishing a primary relation of protection between the embryo and a stranger (in the form of the state or, in Texas, a concerned citizen) excludes the mother of this relation and separates her from it by subjugating her—while she is the only one who can and must decide whether she has the possibility to engage in a long-term care relationship. If she is forced to, the consequences for both her and the child are detrimental.

The discursive tendency of maternal separation instrumentalizes reproductive bodies as vessels instead of relations wherein care for themselves, their children, and their reproductive capacities can take place. This instrumentalization is racialized through a history of colonization, slavery, forced sterilization, and eugenics, and results currrently in higher maternal and neonatal mortality rates.^32–36^ For instance, in the same decade as *Paris Match* featured an early “pro-life” photo series, on the French overseas territory Reunion Island, thousands of Black women were subjected to forced abortions and sterilization without their knowledge or consent.^33^ Francoise Vergès discusses in her book *The Wombs of Women* how this practice, designed to deal with “serious demographic issues” and collect insurance money, was able to continue for years without causing public or ethical outrage nor criminal investigation [33, p. 18]. The discursive power of maternal separation hence *reproduces* the axes of whiteness, Blackness, marginalization, privilege, and the country that one lives in and was born in, among others.^32–34^ The situatedness of these three events in the same time under the power of the same nation-state shows the racial differentiation in biopolitical policy where some people are forced to reproduce while others’ reproductive capacities are destroyed. Both are a clear separation of the reproductive and midwifery relation.

This grip of the state is effectuated through the appropriation of midwives and doctors to the state. Excessive measures in the United States show the force that policymakers deem necessary to sever the relationality and solidarity between mother and midwife or doctor, such as the new law in Texas, or the attempts by other states to make performing an abortion a felony after 6 weeks of gestation.^5,37,38^ Also on the other end of pregnancy, midwives who provide homebirths or follow mothers’ wishes against medical advice are faced with prosecution, and indigenous and traditional birth attendants continue to be juridically pushed out of the domain of childbirth.^39–41^ At a more formative level, students in obstetric training must show assertiveness, power, and responsibility over mothers in order to graduate, which is juridically embedded in mandatory numbers of procedures like episiotomies.^6^ During the whole period of pregnancy, there is a discursive tendency in both the juridical as in the political domain to sever the relation between the maternal and the one who cares for them decreasing the power, autonomy, and freedom of pregnant people which must be constituted relationally.

### The ethical-existential framing of the maternal as maternal–fetal conflict or constraint

Even in countries with policies that guarantee patients’ rights, respectful maternity care is under pressure. Ethnographic research reveals that the expectation that professional experts give objective information remains unfulfilled as these experts are not free from prejudice, their assessment of medical risks is biased, and their relation with the institution they work for is stronger than with the people they care for.^42^ Decisions concerning treatments and interventions are not clearly communicated to the mother, nor is she offered the opportunity to give informed consent.^28,42^ In addition, mothers rarely receive continuous and relational care during pregnancy, childbirth, and postpartum, although the beneficial effects of support and care on maternal and neonatal morbidity and mortality have been proven many times.^43^ The increase in epidurals is not only caused by a higher demand for pain medication but also by the experts’ technocratic values, a fragmented system of maternity care and a lack of continuous relational support.^42^ The absence of both objective information and continuous support attest to the separation of the relationality between mother and midwife, leading, again, to a strategic diminishment of their subjectivity. This effectively results in a shift in priority from the mother’s best interests to what is understood to be the baby’s best interest.^3,10–12^

The focus on the baby’s life as an entity “captured” inside of the mother, instead of relationally intertwined, results in a lack of care for the latter. Mothers express how they are made to feel “less than human,” like a “lump of meat,” and an “obstacle” surrounding the child.^44–46^ Kingma describes these misconceptions as the dominating “fetal container model.”^49^ This model regards the fetus as if it is independently growing within the mother, hence reducing the mother to its container. Katz-Rothman (1986) traces the conceptualization of the individual fetus to the beginning of medical measurements and visualizations of the fetus, which lifted it as a subject out of the mother’s body—recall the *Paris Match* cover story: “The fetus in utero has become a metaphor for ‘man’ in space, floating free, attached only by the umbilical cord to the spaceship. But where is the mother in this metaphor? She has become empty space” [48, see also 49]. With the differentiation from its mother, the fetus is no longer growing within the mother but, rather, within medical discourse. According to Duden,^29^ these developments have “transformed pregnancy into a process to be managed, the expected child into a fetus, the mother into an ecosystem, the unborn into a life, and life into a supreme value.” [29, p. 2]. As such, the maternal in the maternal–fetal organism is established as ontologically secondary to the being of the fetus. In the maternal–fetal container model, maternal separation results in a diminished maternal subjectivity, while the child gets taken up by the obstetric institution as both a subject and the symbolic representation of “Life” that must be defended.

The traditional lack of thought on relationality in Western philosophy works discursively in its understanding of the relational nature of the maternal as an anomaly. Feminist bodies of knowledge that elaborate upon relationality from care ethics (Walker 2007), care-ethical disability studies (Kittay 1999), critical vulnerability studies (Mackenzie & Stoljar 2000; Mackenzie, Rogers & Dodds 2014), and the social practice of identity formation (Lindemann 2014) have not received appropriate attention.^19–21,49,50^ In Western thought, one configuration of the human, that emphasizes identity as differentiation and separation of the individual from others, remains dominant. As Hird (2007) writes:

Insofar as Western societies are dependent upon a notion of freedom prior to constraint and inasmuch as the human body is assumed as clearly and cleanly demarcated from others, then pregnancy, birthing and breastfeeding can only exist as uncomfortable anomalies to human subjectivity.^53^

The lack of impact of feminist philosophies of relationality, and of the experience of fertility, pregnancy and childbirth within philosophy, complicates our understanding of pregnancy and childbirth culturally and politically as the dominant view limits our sense of self, leaving fertility, pregnancy, birth, and early motherhood as an impossibility or problem to our subjectivity that can be easily expropriated. into specialized domains beyond the grasp of pregnant people. The continuous expropriation of relationality from the maternal is ensured by a biopolitical discursive reproduction of mother and child, or woman and “Life”, as separate entities, effectuated in ongoing obstetric reproductive and obstetric violence that is, at its core, a severance of relationality. This dual severance leaves the maternal not only isolated from a community of care, but also alienates them, through the instrumentalization of their reproductive capacities, from their fertility as a possibly existential dimension of the self.

## Re-imagining maternal relationality

### The reproductive relation

In order to take the turmoil of reproduction, giving birth and relational reproductive care seriously, we must dare to re-imagine the relationality and ambiguity of pregnancy and fertility. Following Audre Lorde’s questions: “What are the words you do not yet have?”, we need to question the configuration of the maternal and reproductive relation and ask, freely: What *is* pregnancy? And giving birth? And fertility? And midwifery care? And how do these relations restructure our relation to ourselves and the world?^54^ Lily Gurton-Wachter writes:

How will having a baby disrupt my sense of who I am, of my body, my understanding of life and death, my relation to the world and my sense of independence, my experience of fear and hope and time, and the structure of my experience altogether?^55^

Next to the often-theorized existential dimensions of natality and mortality, reproduction must be re-imagined if we are going to arrive at reproductive justice. The interwovenness of the fetus with the maternal challenges every conception of subjectivity as singular.^56–58^ The modern idea that the subject is enclosed and confined by the skin, an embodied and singular “I,” is no longer valid for a pregnant person. Pregnancy questions these boundaries of the “I,” as the pregnant human does not coincide with their own body in which an other starts to grow. The boundaries of both identities of mother and fetus are therefore opaque and fluid, and relational to the core. Maternal identity is neither one nor two-in-one.

Maternity is not a passive waiting for an already completed other human being that is merely following the course of its fate, but an active awaiting that will go on and on and transforms the maternal together with the formation of the child. During the year of pregnancy, birth, and maternity, the mother’s identity changes fundamentally.^59–61^ The transformation concerns their sense of self (becoming a mother), social status, and activities, but she also transforms on a deeper level from an “I” to the experience of the self as “we.”^62,63^ In pregnancy, there is a constant dynamic of questioning who the other is, how the other is, of interpreting and circumventing the child whose limbs are formed in dialogue with the maternal movements of nurturance, of healing, of making milk.^50,64^ The maternal relation is genealogy and generativity: both are embedded in past and future generations, and diachronically the newborn is embedded in “his/her generation.”^65^ Relationality in pregnancy is both spatial and temporal: throughout pregnancy the maternal body is reshaped, inflated, making space for, and increasingly co-possessed by the other-in-the-self.^53,66,67^

Where natality and mortality individualize, pregnancy and fertility make us plural, by carrying the possibility of the natality of the other. The possibility of the other’s natality is constitutive for our fertility. This means that a relational view of pregnancy and birth forms the foundation of two existential structures that cannot be separated: the specific natality of the fetus that structures fertility and pregnancy on the one hand, and the fertility that enables the specific natality of this fetus on the other hand. The possibility of something new lies less in natality, as Arendt^52^ has argued, but is located in the relationality of reproduction, as a sympoetic productive intertwining of fertility, natality, community, and care.

### The midwifery relation

A relational form of abortion and midwifery care would consist of long-term individual or communal relation-building, that allows for freedom of choice, in-depth conversations on pregnancy, birth, and the needs of mother and child after birth to make another ethical, existantial and communal consciousness possible through experience, re-imagination, receptivity, and spirituality.

Only tailor-made care can hope to attune to the concrete person. The thought, decisions, and subjectivity of the maternal can be seen as primarily structured within the specificity of their circumstances: it is always about *this mother, this child,* *in this world*. As such, pregnancy and childbirth should be approached intersectionally.^69^ This requires diversity, cultural humility and conversations about beliefs and considerations concerning morally good and meaningful maternity practices and courses of action with maternity care workers.

*“Everybody is some mother’s child*,*”* writes care ethicist and disability philosopher Eva Feder Kittay.^19^ This shared human condition of a bodily and dependent origin is the foundation of human equality, rather than any individual condition or capacity (such as rationality or autonomy). Within her famous quote one can also change one’s perspective: it takes both a mother *and a midwife caring for a mother* to come into existence. Each human being owes his or her existence to a person who got pregnant, has experienced that pregnancy positively or negatively, has felt this unborn being, fed it and eventually gave birth to it, *and* those who cared for her. Throughout this process all three—mother, midwife, and child—can transform and be given, like in the case of Annie Ernaux, a world. Opening the imaginary through philosophy, art, activism, and, most importantly, care, can help us to reconceive relationality for reproductive justice.

## Conclusion

We have discussed the discursive structural tendency of what we call “maternal separation” as a cause of reproductive and obstetric violence. This maternal separation racializes and instrumentalizes the reproductive subject and consists of a severance of the double relationality that constitutes the maternal: (1) The reproductive relation (the relation between the maternal and the potential child) and (2) the midwifery relation (the relation between mother and midwife, or the community that cares for the maternal). We have identified maternal separation in two domains, the juridical-political and the ethical-existential. This separation ultimately leads to the expropriation of the relationalities that are constitutive of the maternal thereby violating, isolating, alienating and instrumentalizing the reproductive subject. For reproductive justice and emotionally and physically safe maternity care to become possible, both the reproductive and the midwifery relation must be radically re-imagined. With this contribution we hope to underscore the need for care ethics because of its traditional focus on relationality, and relational autonomy in maternity care, as well as to lay bare what inhibits the relationalities necessary for a truly care-ethical praxis. Furthermore, it aims to shine another light on debates concerning abortion and childbirth leading to ethical questions and problematics that differ from those more commonly raised, centering on interwovenness relationality, community and solidarity. Consequently, midwifery needs to start including abortion more prominently in its philosophy and re-imagination of care to ensure relationality not only surrounding childbirth, but also surrounding abortion.

## Notes

1 As we have argued elsewhere, we choose to use the terms “mother” and “maternity” as social economical gendered subject categories, not as an essentialist sex differentiation.^6–8^ This does not mean that people with a uterus who do not identify as “mothers” are not victims of reproductive or obstetric violence, on the contrary. We understand maternal separation to be a form of violence against all reproductive people (those who identify as mothers and those who do not) as it consists of the severance of relationalities that makes up an existential caring relation present as a capability or possibility of reproduction, a structure of being we identify as “maternal.”

2 For an overview, see: https://reproductiverights.org/worldabortionlaws

3 In Oslo, Norway, thousands of people protested against plans to restrict abortion law in 2018, but a restricted abortion law passed parliament in June 2019 (News in English, 14 June 2019). In Poland, since 28 of October 2020, abortion remains legal only in case of rape, incest, or when the life of the pregnant person is in danger. Abortion in case of fetal defects was ruled to be unconstitutional. In Iceland, major protest did not prevent the passing (April 2019) and going into effect (September 2019) of a restricted new abortion law (The Reykjavík Grapevine, 2 September 2019). In other countries, an explicit demographic policy has been introduced as part of populist politics. In Hungary, for example, women are rewarded for having many children. They are exempt from income tax for life, after having a fourth baby. Prime Minister Orbán explicitly makes a connection between Hungarian reproduction and xenophobic and homophobic defense of Hungarian culture (The Guardian, 10 February 2019).
